# Harmonized disposable income dataset for Europe at subnational level

**DOI:** 10.1038/s41597-024-03138-x

**Published:** 2024-03-21

**Authors:** Mehdi Mikou, Améline Vallet, Céline Guivarch

**Affiliations:** 1 Université Paris-Saclay, AgroParisTech, CNRS, Ecole des Ponts ParisTech, Cirad, EHESS, UMR CIRED, 94130 Nogent-sur-Marne, France; 2 Université Paris-Saclay, CNRS, AgroParisTech, Ecologie Systématique et Evolution, 91190 Gif-sur-Yvette, France

**Keywords:** Economics, Geography, Environmental economics, Sustainability

## Abstract

In recent decades, detailed country-level estimates of income and wealth have become widely available and inform us about the evolution of inequality between and within countries. But a substantial portion of these available datasets lack sub-national geographical information, precluding the exploration of the spatial distribution and evolution of inequalities within countries. We present here a new dataset of disposable income for Europe at the subnational level. It has been compiled from existing income data (gross income, gross earnings, equivalised income, etc.) published by national statistical institutes at different geographical levels. We used linear regressions and numerical operations to estimate disposable income from other available socio-economic statistics (e.g. household size, tax rates). We developed a harmonization and adjustment procedures to ensure of the consistency of statistical units, income indicators, costs of living and inflation. The dataset covers 42 European countries distributed over more than 120,000 geographical entities on the 1995 to 2021 period (most of the data being available for the 2010–2020 decade). This new dataset opens avenues for investigating the links between income inequality and other socio-economic or ecological processes.

## Background & Summary

Since 1980, within-country inequalities have started to rise after decades of decrease during the 20^th^ century^1^. These analyses of inequities are made possible thanks to the availability of detailed income and wealth datasets. At the global level, the World Inequality Database (https://wid.world/) provides time-series of average income for each centile along the income distribution. While very informative to track the evolution of income inequalities over time, this dataset poorly informs on the spatial distribution of inequalities within countries, as they rather focus on vertical inequalities (i.e. inequalities among individuals). Spatial inequalities are much more difficult to capture because of the limitation of fine-resolution datasets and statistical data.

At the global level, several studies have developed high-resolution datasets of economic activity. For instance, Gennaioli *et al*.^2^ have constructed a database of subnational regional incomes in 110 countries. Smits & Permanyer^3^ have collected subnational gross national income per capita to build a subnational index of human development. And finally Wenz *et al*.^4^ have produced a timeseries of subnational database of gross regional product per sector between 1960 and 2020. These 3 studies have used in Europe income data published annually by Eurostat (the statistical office of the European Union) at the NUTS2 level (basic regions for the application of regional policies) or NUTS3 level (small regions for specific diagnoses). Although finer than national data, the NUTS2 and NUTS3 levels are still quite coarse (average size of NUTS2 units is approx. 18,000 km^2^), and does not allow for an assessment of local spatial inequalities. At a much finer resolution, several gridded gross domestic product (GDP) datasets have recently been developed based on existing regional GDP data^5–7^. However, regional GDP can only represent regional inequalities to a limited extent, as it does not take into account the redistribution made possible by taxes and transfers. Moreover, regional GDP in Europe may be skewed in favour of regions with net commuter inflows and against regions with net commuter outflows^8^.

However, national statistical institutes (NSIs) often publish sub-national data (below NUTS2 level) about income. These estimates are produced at different levels of administrative units (AUs), and refer to various stages in the distribution of income and earnings (gross income, gross earnings, equivalised income, etc.). They are calculated using different statistical indicators (mean, median), and different statistical units (household, individuals, workers, etc.). Moreover, some AUs are not constant over time, which requires considering their geographic evolutions, that can happen through fusions or splits of one or multiple AUs.

We present in this paper a new dataset of harmonized disposable income for Europe, using the finest information distributed by NSIs. According to the European System Accounts^8^, disposable income is defined as the sum of net operating surplus/mixed income, compensation of employees, balance of property income, social benefits received including old-age pensions, from which is deducted taxes on income/wealth and compulsory social contributions. Disposable income has the advantage of considering all sources of income earned by households and to account for redistribution schemes within countries. When NSIs did not provide directly this indicator, it was estimated using linear regressions as well as other available indicators such as taxation rates. Finally, to account for the size of the population within AUs, we calculated per capita disposable income (i.e. the sum of disposable income of all households divided by the total resident population of the AU).

The resolution and the quality of NSIs’ data also varied considerably across countries. For example, the average size of AUs ranged from 1.5 km² in Belgium to 50,000 km² in Bosnia and Herzegovina. Very high-resolution datasets released in countries such as France and Spain suffered from incompleteness (presence of NA values). The final dataset covers 42 European countries - distributed over more than 120,000 AUs - over the period 1995–2021. This temporal coverage is not homogeneous between countries and is mainly centred on the 2010–2020 decade. With this dataset, we open new avenues for socio-economic modelling in Europe, which can contribute to the work of various research communities (vulnerability to climate change, poverty mapping, etc.).

## Methods

**Fig. 1:**
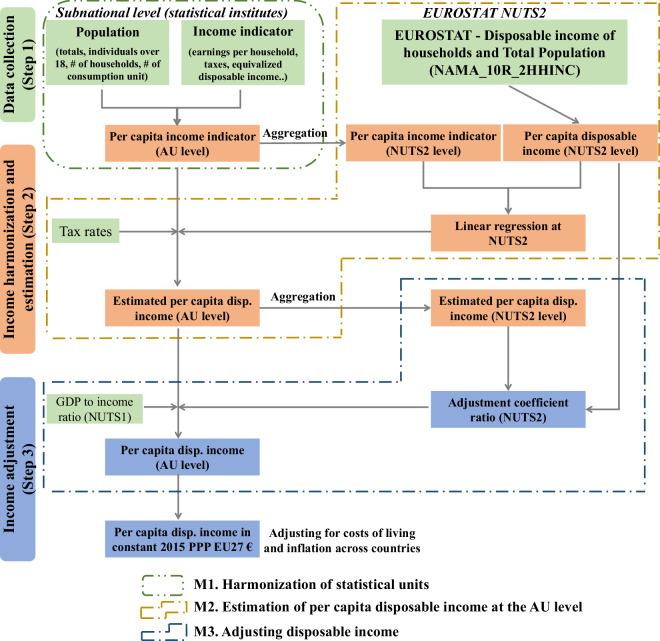
Workflow for developing the European income database.

The database was developed in a three-step workflow summarized in Fig. 1. The first step consisted in collecting income and other auxiliary data from NSIs. In the second step, collected income (from step 1) was harmonized and then used as an input for the estimation of disposable income. The third step of the workflow ensured that the estimated income (from the previous step) was consistent with other databases and that incomes were comparable across countries. For steps 2 and 3, we developed 2 different approaches depending on whether the country was covered or not by Eurostat statistics at NUTS2 level. To report on the quality of the data provided for each country, we developed a country-level quality score based on 6 attributes of the workflow.

The harmonized dataset was created using Python 3.6.13^9^ and QGIS 3.16.11^10^. Country-specific folders containing a Jupyter Notebook and description files are accessible in open access at the Zenodo repository^11^. Input data can be found in the Recherche Data Gouv replication repository^12^. Description files contain links to all governmental and third party data sources used and all steps to convert NSIs’ income to disposable income. Table S1 (in Supplementary Information) also provides a summary of all the sources used to download input data. For most countries, input data was accessible in open access (to download the public records for specific years, users can either: (1) visit the websites listed in Table S1 under the column “Name of download income variable and download url” and search for the listed variable; (2) follow the detailed instructions provided in the country description files or in the Jupyter Notebooks^11^). The raw data for Belgium, Hungary, Czech Republic, Austria and Italy was supplied by the “Directorate for Employment, Labour and Social Affairs” (https://www.oecd.org/els/) of OECD^13^ as part of a collaboration with the authors of this work. Others wishing to repeat the work or perform similar analyses should approach the authors of the OECD study^13^ directly. Similarly, users should contact the “Economic council of the Labour Movement” of Denmark (https://www.ae.dk/kontakt) to get data for this country.

### Collecting income and socio-demographic data from national statistical institutes (Step 1)

The database was compiled from NSIs at the finest AU level publicly available. It covered 42 European countries, from 1995 to 2021 (Table 1). The income data collected across countries correspond to different stages of incomes and earnings (net earnings, gross income, disposable income etc.), and was aggregated at the AU level using different statistics (average, equivalised average, or median). In total, the dataset contains about 120,000 AUs, whose boundaries are presented in Supplementary Information, Figure S1.

**Table 1:** Summary of income data used in the study.

Country	Indicator	Year	Administrative units	Harmonization of statistical units (M1)	Estimation of income (M2)	Adjusting disposable income (M3)
**Albania**	Household consumption expenditure	2007-2009-2014 to 2020	12 counties	Household	Taxation	GDP to Income ratio
**Andorra**	Mean income	2018 to 2020	Country level	Population	—	—
**Austria**	Net income	2008 to 2019	2747 municipalities	Total value	Linear regression	Eurostat NUTS2
**Belarus**	Gross income	2016 to 2021	7 regions	Total value	Taxation	GDP to Income ratio
**Belgium**	Total net taxable income	2005 to 2018	~ 19700 statistical sectors	Total value	Linear regression	Eurostat NUTS2
**Bosnia-Herzegovina**	Av. net and gross earnings	2008 to 2020	Country level	Employed population	—	GDP to Income ratio
**Bulgaria**	Total gross income per capita	2008 to 2021	28 districts (NUT3)	Population	—	Eurostat NUTS2
**Croatia**	Net earnings	1994 to 2020	21 counties	Employed population	—	Eurostat NUTS2
**Cyprus**	Net Income	2015	5 districts	Household	—	Eurostat NUTS2
**Czech Republic**	Av. disposable income	1995 to 2020	14 regions (NUTS3)	Population	—	Eurostat NUTS2
**Denmark**	Eq. disposable income	2010 to 2019	2220 parishes	Equivalised	—	Eurostat NUTS2
**Deutschland**	Tax income	1995 to 2018	~ 4400 municipalities	Total value	Linear regression	NUTS3
**Estonia**	Gross earnings	2013 to 2020	79 municipalities	Employed population	Linear regression	Eurostat NUTS2
**Finland**	Av. disposable income	2012 to 2017 – 2019 to 2020	3030 postal codes	Population over 18 years old	—	Eurostat NUTS2
**France**	Median eq. disposable income	2012 to 2018	~ 35000 communes	Eq.	Linear regression	Eurostat NUTS2
**United Kingdom**	Gross disposable income	1997 to 2017	~ 374 local authorities	Population	—	GDP to Income ratio
**Greece**	Av. disposable income	2000 to 2020	13 regions (NUTS2)	Population	—	Eurostat NUTS2
**Hungary**	Total taxable income	2009 to 2020	20 NUTS3	Total value	——	Eurostat NUTS2
**Iceland**	Av. disposable income	1998 to 2020	69 municipalities	Taxpayer	—	GDP to Income ratio
**Ireland**	Median gross income	2016	3409 electoral divisions	Household	Linear regression	Eurostat NUTS2
**Italy**	Taxable income	2012 to 2018	8000 municipalities	Total value	Linear regression	Eurostat NUTS2
**Kosovo**	Net av. wage	2012 to 2020	Country level	Employed	—	GDP to Income ratio
**Latvia**	Mean disposable income	2004 to 2021	6 regions	Population	—	Eurostat NUTS2
**Liechtenstein**	Median gross wage	2006 to 2020 (every 2 years)	12 municipalities	Employed population	—	Country level
**Lithuania**	Av. disposable income	2014 to 2020	10 NUTS3	Population	—	Eurostat NUTS2
**Luxembourg**	Median salary	2015	105 communes	Employed population	—	Eurostat NUTS2
**Macedonia**	Disposable income	2010 to 2020	Country level	Household	—	GDP to Income ratio
**Malta**	Disposable income	2014 to 2020	Country level	Total value	—	—
**Moldova**	Disposable income	2006 to 2018	4 regions	Population	—	GDP to Income ratio
**Montenegro**	Mean eq. disposable income	2013 to 2020	Country level	Equivalised	—	GDP to Income ratio
**Netherlands**	Av. eq. income	2011 to 2019	355 municipalities	Equivalised	Linear regression	Eurostat NUTS2
**Norway**	Median after-tax income	2005 to 2021	400 municipalities	Taxpayers	—	Eurostat NUTS2
**Poland**	Av. gross wages and salaries	2002 to 2020	380 counties	Employed population	Linear regression	Eurostat NUTS2
**Portugal**	Gross income less income tax	2015 to 2019	308 communes	Population	Linear regression	Eurostat NUTS2
**Romania**	Av. net earnings	2008 to 2021	42 counties	Employed	Linear regression	Eurostat NUTS2
**Serbia**	Income in money	2006 to 2021	4 regions	Household	Taxation	GDP to Income ratio
**Slovak Republic**	Disposable income	2010 to 2021	7 regions	Population	—	Eurostat NUTS2
**Slovenia**	Net income	2014 to 2020	200 local administrative units	Population	—	Eurostat NUTS2
**Spain**	Net Income	2015 to 2019	~ 36000 administrative units	Population	Linear regression	Eurostat NUTS2
**Sweden**	Disposable income	2000 to 2020	290 local administrative units	Population	—	Eurostat NUTS2
**Switzerland**	Net income	2010 to 2019	2008 communes	Total value	Taxation	Country level
**Ukraine**	Disposable income	2002 to 2021	27 oblasts	Population	—	GDP to Income ratio

In the column Harmonization of statistical units (M1), “Population” means that income was collected in per capita terms using the total population of the corresponding AU; “Household” that the total income was divided by the number of households of the AU; “Total value” that income was provided in aggregate terms by the NSI and “Equivalised” that household income took into account the differences in size and composition of households. “Av.” stands for average and “eq.” for equivalised.

Income data was always distributed by NSIs in a tabular format, which we matched with geographic data for AU boundaries. When available, specific administrative boundaries were used for each income year. When not, the geographic data available for the nearest year was manually modified to incorporate changes that might have happened to AU boundaries, such as fusions between two municipalities. These operations on AU boundaries are described in detail in the Jupyter Notebook associated to each country and were mainly performed using the GeoPandas^14^ 0.9.4 and Pandas^15^ 0.25.3 packages.

For harmonization purposes, we also collected other auxiliary data (such as total population, employed population, number and size of households…) from the NSIs at the same AU level as income data. At the NUTS2 level, we collected per capita disposable income and total population (can be retrieved from total and per capita disposable income) from Eurostat (https://ec.europa.eu/eurostat/databrowser/view/NAMA_10R_2HHINC/default/table?lang=EN).

### Harmonizing national statistical institutes’ income data and estimation of disposable income (Step 2)

To create the European dataset of per capita disposable income, a harmonization procedure was developed (M1 in Fig. 1). It consisted in the harmonization of all income indicators to a common statistical unit (also called unit of observation or measurement, for which the income information was collected), namely the total population of the AU. Then, the estimation of disposable income method (M2 in Fig. 1) allowed to convert incomes corresponding to different stages of earnings and income (pre-tax income, net earnings…) to disposable income.

### Harmonization of statistical units (M1)

The harmonization procedure was performed through the use of auxiliary variables collected at step 1. In addition to converting collected AU income to the same statistical unit, we also performed for some countries an income aggregation to a higher geographical unit (NUTS2 or country-level). In Norway for example, the average NSIs’ income indicator was originally computed on the basis of the number of persons over 18 years old. After collecting total population and population over 18 years old in each AU, we have used a population weighted average to aggregate the income of multiple AUs and get the income value of their corresponding NUTS2 unit, using Eqs. 1 and 2:

1 $$inc\_ca{p}_{AUi}=\frac{inc\_taxpayer{s}_{AUi}\ast num\_taxpayer{s}_{AUI}}{populatio{n}_{AUi}}$$

2 $$inc\_ca{p}_{N2j}=\frac{{\sum }_{AUi\in N2j}inc\_ca{p}_{AUi}\ast populatio{n}_{AUi}}{{\sum }_{AUi\in N2j}\,populatio{n}_{AUi}}$$

where inc_cap_Aui_ refers to per capita income, inc_taxpayers_Aui_ refers to the average income per taxpayer, num_taxpayers_Aui_ the number of taxpayers, population_Aui_ the total population within AU I and inc_cap_N2j_ the population weighted average income in the NUTS2 j in Norway.

For the Netherlands and Denmark, whose NSIs release average standardized income (it refers to disposable income adjusted for differences in household size and composition), we first calculated the average size of households (persons per households) within each AU, and then we converted them to per capita disposable income (inc_cap_Aui_) using equivalence factors (they reflect economies of scale in a household), as described in Eq. 3:

3 $$inc\_ca{p}_{AUi}=\frac{standardized\_in{c}_{AUi}\ast num\_household{s}_{AUI}\ast equiv\_facto{r}_{AUi}}{populatio{n}_{AUi}}$$

where standardized_inc_Aui_ is the average standardized disposable within AU i, num_households_Aui_ is the total number of households, population_Aui_ the total resident population and equiv_factor_Aui_ the calculated equivalence factor related to the average size of households. We have then applied Eq. 2 to obtain an indicator at the NUTS2 level. We implicitly assumed here that average standardized income is equal to the average income per household divided by the equivalence factor of the average size of the household within the AU i.

### Estimation of per capita disposable income at the administrative unit level (M2)

#### Countries covered by Eurostat

When not directly available, disposable income at AU level was then estimated using a linear regression developed at NUTS2 level (taking advantage of income data provided by Eurostat), that we then applied at the finest AU level. The linear regression allowed us to estimate for each country separately the relationship between disposable income and the income indicator distributed by the NSI, and to correct for the use of different methodologies in the collection of income between Eurostat and NSIs. For Germany, the linear regression was developed at NUTS3 level as finer resolution information was available. The linear regression was performed using the LinearRegression function from the Python scikit-learn package^16^.

The regression was developed on a yearly basis for countries having at least 8 NUTS2 units, the minimum sample size that allows to perform a regression^17^. Countries not fulfilling this condition were not concerned by this step. No harmonization was performed in Sweden, Greece and the Czech Republic, as the data distributed by the NSIs corresponded exactly to the data distributed by Eurostat at NUTS2 level.

**Fig. 2:**
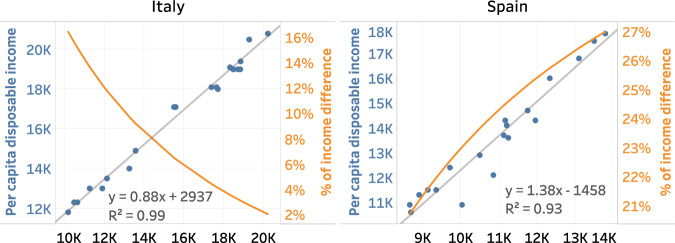
Regression of per capita disposable income on NSIs’ income for Italy and Spain in 2015. Each point represents NSIs’ income and disposable income of a NUTS2 or NUTS1 area in Italy and Spain. The grey line is the trend line (with its associated equation). The orange curve represents the difference in income induced by the conversion to disposable income. For example, in Italy, an income of €9,000 increases by about 16% when converted into estimated disposable income, while an income of €17,000 increases by only 2%.

In Italy, disposable income was estimated from per capita post-tax income at the NUTS2 level, using a linear regression (Fig. 2). Compared to disposable income, social benefits received by households are not included in the post-tax income. The linear regression performed for Italy in 2015 showed a high R² value (0.99), which means that most of the variation in disposable income can be explained by a variation in taxable income. In Spain, disposable income was estimated from per capita net income using a linear regression that also exhibited good results (R²=0.93).

The parameters of the regression in these 2 countries lead to different levels of estimated disposable income (Fig. 2). In Italy, the regression leads to levels of estimated disposable income higher than post-tax income for all income levels, with low income areas benefiting proportionally more than high income areas. In Spain, estimated disposable income is also higher than net income, with high income areas benefitting more from the regression compared to low income areas. These examples highlight some characteristics of the estimation of disposable income with a linear regression.

#### Countries not covered by Eurostat

In Switzerland, where only net income was provided by the NSI, we used tax rates available at the cantonal level – that depend on the religious affiliation, the income level and the number of persons in the households – to calculate disposable income. In Belarus, a constant tax rate was used to account for missing detailed information about taxes. Applying tax rates to average income at the AU level and not at the household level is a limit of our approach, that we cannot avoid when household income data is not available. Indeed, averaging post-tax income of individual households would probably lead to a different result compared to applying a unique tax rate to the average income of households.

Table S1 (in Supplementary Information) provides a country-level description of the methodology used for the estimation of disposable income.

### Income adjustment (Step 3)

#### Adjusting disposable income (M3)

In many cases, the estimation of disposable income was limited, either because some components of disposable income (such as pensions or social benefits received by households) were not available or because the number NUTS2 units was not sufficient (below than 8) to perform a linear regression. In a third and last step, we therefore adjusted the disposable income estimates obtained at M2 to match other databases. Prior to the adjustment of per capita disposable income, incomes for countries outside the euro area were converted from national currency to Euro using Eurostat conversion rates (https://ec.europa.eu/eurostat/databrowser/view/ERT_BIL_EUR_A).

#### Countries covered by Eurostat

Disposable income estimates obtained by linear regressions for each AU were aggregated at the NUTS2 level using a population-weighted average. By computing the ratio between these estimates and the Eurostat data of disposable income at NUTS2, we were able to uniformly adjust AU-level disposable income. More specifically, each AU within a NUTS2 region was multiplied by this ratio. This step allowed us to ensure consistency of AU income distribution across NUTS2 units within a country. In Greece, income was not adjusted as it was directly collected from Eurostat NUTS2 disposable income.

#### Countries not covered by Eurostat

For the countries not covered by Eurostat, the adjustment was based on Eq. 4 that assumed that the country level per capita disposable income can be retrieved from its GDP, the average disposable income and GDP of the EU27 (refers to the 27 European Union countries in 2020 following Brexit) countries:

4 $$\frac{disp\_in{c}_{i}}{disp\_in{c}_{EU27}}=\frac{GD{P}_{i}}{GD{P}_{EU27}}$$

where disp_inc_i_ is the per capita disposable income of country i, disp_inc_EU27_ is the average disposable income of EU27 countries, GDP_i_ is the per capita gross domestic product of country i and GDP_EU27_ the average gross domestic product of EU27 countries. GDP data was retrieved from Eurostat (https://ec.europa.eu/eurostat/databrowser/view/NAMA_10_PC/) or from the World Development Indicators database (https://data.worldbank.org/indicator/NY.GDP.PCAP.PP.CD). By adjusting income at the country level, this step ensures that income levels are consistent across countries, but might bias the comparison within countries. As some components of disposable income (such as social transfers) were missing from the collected income indicator definition, differences in adjusted income between AUs within a country might hence not truly reflect the differences in disposable income.

Income for countries such as Switzerland, Liechtenstein, Malta and Andorra – which are considered tax havens – were not adjusted using Eq. (4) because GDP flows largely exceed income flows for these countries^18^. In Switzerland and Liechtenstein, income was adjusted using country-level disposable income estimates published by NSIs. In Andorra and Malta, no adjustment was performed as no external country-level source of disposable income was found.

### Adjusting for costs of living and inflation across countries

To remove the effects of price differences across countries, all income estimates were converted to a single currency 2015 PPP EU27 € (EU27 corresponds to the 27 countries of the European Union after the Brexit in 2020) using Eurostat price level indices (https://ec.europa.eu/eurostat/databrowser/view/prc_ppp_ind/default/table?lang=en). To compare incomes over time, we used harmonised indices of consumer prices (https://ec.europa.eu/eurostat/databrowser/view/PRC_HICP_AIND/default/table?lang=en) to remove country-level inflation effects (using 2015 as the base year). National indices of consumer prices and purchasing power parity conversion factor from the World Development Indicators (https://data.worldbank.org/indicator/PA.NUS.PPP) were used when countries were not covered by these 2 databases.

To convert nominal income to 2015 PPP EU27 €, we first converted the nominal income of an AU located in country c in euros in year 2015 (AU_income_€_nominal,2015,c_) to an income in purchasing power parities using price_level_indices_c,2015_ of country c in relation to EU27 for year 2015 (Eq. 5). To remove inflation effects across years, we then multiply the income in purchasing power parities by the AUs’ growth rate of constant income in local currency between the year y and 2015 (Eq. 6).

5 $$AU{\rm{\_}}incom{e}_{EU27PPP2015,y,c}=\frac{AU{\rm{\_}}income{\rm{\_}}eur{o}_{nominal,2015,c}}{price{\rm{\_}}level{\rm{\_}}indice{s}_{c,2015}}(1+AU{\rm{\_}}income{\rm{\_}}growt{h}_{lcu,2015,y})$$

6 $$AU{\rm{\_}}income{\rm{\_}}growt{h}_{lcu,2015,y}=\left(\frac{AU{\rm{\_}}income{\rm{\_}}lc{u}_{constant,y,c}-AU{\rm{\_}}income{\rm{\_}}lc{u}_{constant,2015,c}}{AU{\rm{\_}}income{\rm{\_}}lc{u}_{constant,2015,c}}\right)$$

This approach used for costs of living and inflation adjustment is the one recommended by the International Monetary Fund^19^. Other approaches have been proposed, consisting for instance in applying price level indices (to control for purchasing power between countries) and consumer price indices (to control for inflation between years)^20^. Contrary to the methodology used here, these alternative approaches do not allow to reconstitute the values of GDP per capita in PPP constant 2017 international $ from the World Bank data portal (https://data.worldbank.org/indicator/NY.GDP.PCAP.PP.KD). Furthermore, they rely on annual rates of price level indices that are subject to a high degree of uncertainty^21^.

Using national consumer price indices in combination with subnational income data do not allow to capture the contrasted evolution of prices across different regions of a country. This limitation could partially be resolved by the use of subnational price indices, but these are rarely provided by NSIs.

### Missing information

In countries that publish very high-resolution data (such as France, Spain and Italy), often population or income data were missing from the tabular files distributed by NSIs (Table 1). To obtain a complete dataset, NA values were replaced either with the average value of population or income in the neighbouring AUs or with the value from a higher administrative unit if no neighbouring AUs are recorded. An “Inc_Q” column was added to the final database to allow users to distinguish between originally missing data from the data retrieved from NSIs (identified by “m”) and non-missing data (coded as “s”).

### Country quality score methodology

To assess the quality of the data we generated for each country, we constructed a country-level score based on 6 components of the data distributed by national statistical institutes (NSIs). For each characteristic, we attributed a value of 1 (low quality), 2 (average) or 3 (high quality) according to the following rules:

- **Income indicator distributed by NSI:** A value of 3 was given to countries where disposable income was directly available. For any other type of income, a value of 2 was attributed. When the indicator published by NSIs did only account for one source of income (earnings, salary…), a value of 1 was given.
- **Statistical unit:** When data was distributed in per capita terms using total population as the statistical unit, we attributed a score of 3. A value of 1 was given to countries using equivalised income that takes into account the differences in a household’s size and composition. The value of 2 was given to any other statistical unit. This scoring reflects the additional datasets needed to convert the collected indicator to a common statistical unit.
- **Share of NA values (i.e. completeness of the income data):** For each country, the share of administrative units with NA values was computed and countries were ranked from 1 to 3 using terciles.
- **Size of administrative units (i.e. average area):** We calculated the average area of administrative units over the total area of the country. Countries were then ranked from 1 to 3 using terciles.
- **Availability of the data over multiple years (i.e. temporal coverage):** When data was available for less than 10 years, a value of 1 was given. Between 10 and 18 years, a value of 2 was attributed. Countries recording more than 19 years of income data were given a value of 3.
- **Adjustment coefficient (i.e. performance of the adjustment procedure**): Absolute values of average country adjustment coefficients were used to rank countries using terciles.

The data quality score is a weighted average of the value of the 6 components. Adjustment coefficient and average area components were given a weight of 3 while other components were given a weight of 1. When no adjustment was performed (see M3), the data quality score was computed using the other 5 components. The 6 components of the quality score are indicative of the relative quality of the data and can not fully describe the various specificities of the different countries. For example, countries in Northern Europe having large AUs in low density areas are penalized by the “Size of administrative units” component.

## Data Records

Data records are composed of 2 datasets: (1) a replication dataset which compiles the original data sources^12^; (2) the harmonized dataset resulting from the methodology presented in this article^22^. Both datasets were generated for European countries and are accessible under the CC-BY licence. All data sources compiled in the first dataset are presented in Table S1. The harmonized dataset is distributed as a Geopackage file in the WGS84 latitude/longitude coordinate system (EPSG code 4326), for each year. The variables included in this file are listed in Table 2. In addition to income indicators and country-specific auxiliary data (AU name or code, associated NUTS2 code), Gini coefficient^23^, a measure of income inequality, was also collected either at the same AU level (when available) or from a higher AU. When obtained from a higher AU, the name or code of the higher AU was recorded under the field “Entity”.

**Table 2:** Description of the information provided for each polygon of the final income dataset.

Attribute name	Data type	Description	Example
Year	Numeric	Year of the data	2015
AU_name	Text	Name of the AU	“POLI”
AU_code	Text	Code of the AU	“058078”
NUTS2	Text	Code of the related NUTS2 area	“ITI4”
ISO	Text	Country ISO3 code	“ITA”
Disp_Inc_PPP_15	Numeric	Per capita disposable income in 2015 PPP EU27 €	11,825.1
Disp_Inc_PPP	Numeric	Per capita disposable income in PPP EU27 €	15,000
Inc_Q	Text	Availability of initial income data	“s”
Population	Numeric	Total population of the administrative unit in persons	2,374
Gini	Numeric	Gini coefficient of the Administrative unit	0.31
Gin_Q	Text	Availability of Gini coefficient at the AU level	“m”
Entity	Text	Higher administrative unit from which Gini coefficient is retrieved	“ITI43”

**Fig. 3:**
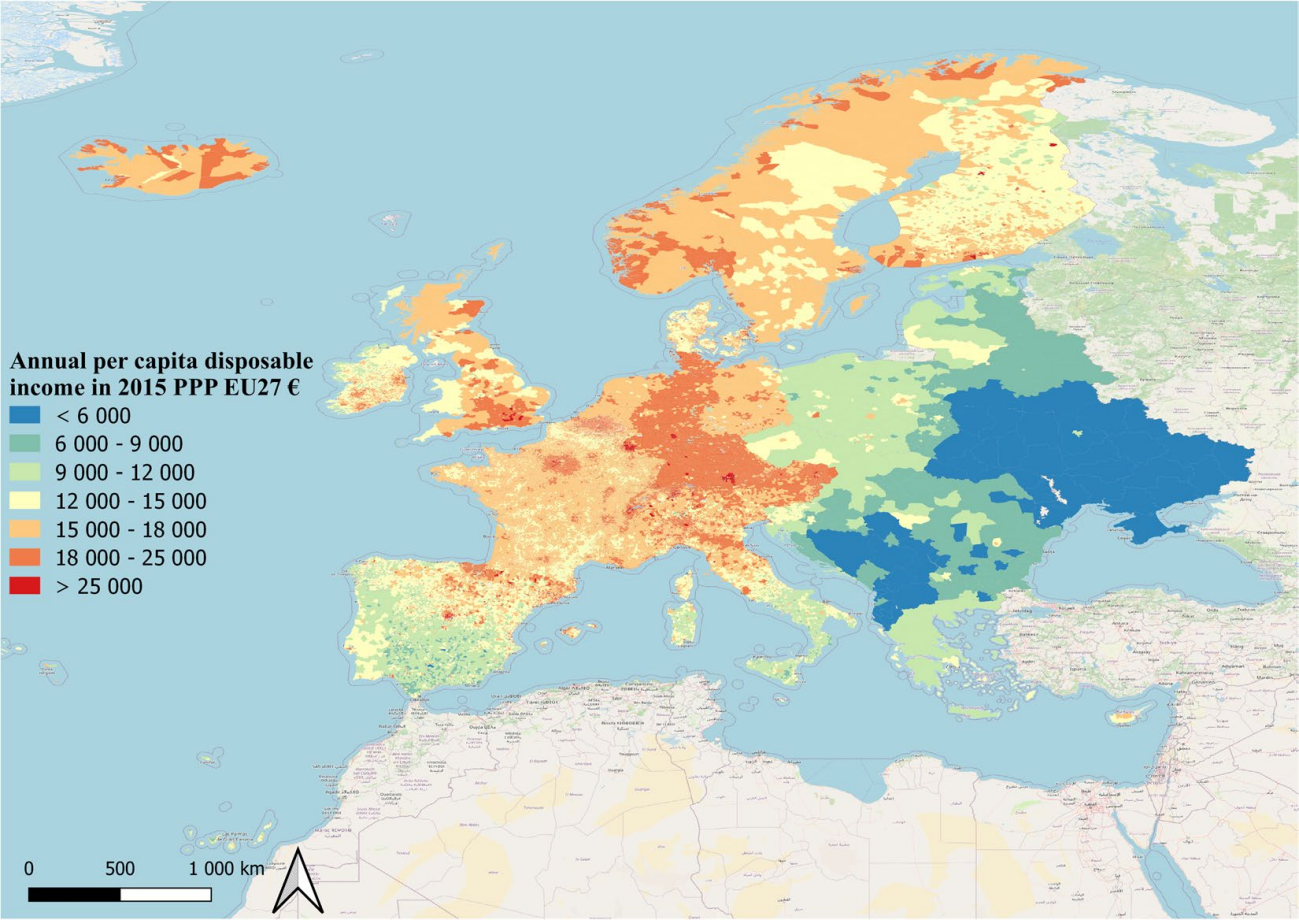
Per capita disposable income map in 2015.

Income distribution in 2015 in European countries showed a strong longitudinal and latitudinal gradient, with western and northern countries having higher incomes than southern and eastern countries (Fig. 3). The high resolution of the dataset facilitated the visualization of intra-country patterns, with higher incomes around capital cities. The size of U in countries belonging to the European Union were much lower compared to eastern Europe countries.

## Technical Validation

The validation of our approach was twofold: (1) assessing the accuracy of estimated disposable income for countries covered by Eurostat and (2) relevance of income and GDP ratios to adjust for non-Eurostat countries.

### Estimation of disposable income accuracy for countries covered by Eurostat

**Fig. 4:**
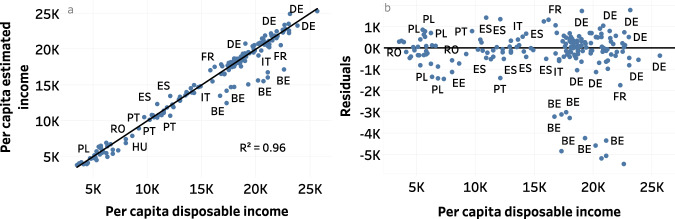
Validation of estimated disposable income. Each point represents a Eurostat NUTS2 region, except for Germany where NUTS3 data is used. (**a**) Comparison between estimated disposable income and Eurostat NUTS2 data. (**b**) Residuals of disposable income estimates: a positive value corresponds to an overestimation and conversely. In figure a, the black line represents the identity line.

To validate our data, we compared estimated disposable income aggregated at the NUTS2 level with disposable income obtained from Eurostat for all countries. The overall accuracy of our estimates was good, with a coefficient of determination (R²) of 0.96, a relative root mean square error of 8% (meaning that our predictions were off by 8% on average), and a negative bias of € 239 (Fig. 4a). This negative bias means that the estimated disposable income was on average lower than Eurostat data for disposable income at NUTS2 level (Fig. 4b).

### Adjustment relevance for non-Eurostat countries

**Fig. 5:**
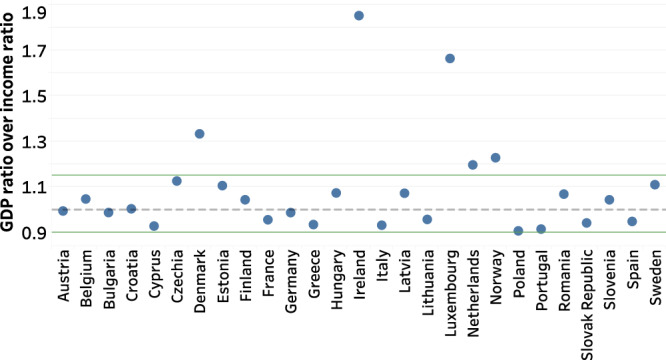
Assessing the validity of income adjustment for countries not covered by Eurostat.

We compared the two sides of Eq. 4 to assess the relevance of using a GDP / income ratio to adjust income in countries not covered by Eurostat. For most countries covered by Eurostat, this ratio ranged between 0.9 and 1.15, which supports its validity (Fig. 5). Luxembourg and Ireland are clear outliers, with a GDP ratio (GDP of the country in relation to the average GDP of the EU27) that far exceeds the income ratio (income of the country in relation to the average income of the EU27), which can be explained by the unequal flows between income and GDP in tax havens^18^. In Ireland, where many foreign companies have shifted their profits, a higher level of profits-to-wage ratio has been observed for these firms when compared to other countries^18^. As these profits enter in the computation of the GDP, the level of GDP end-up being inflated relatively to other countries.

Each dot represents the ratio of GDP (country GDP over average EU27 GDP) over the income (country income over EU27 average income) ratio in 2015. Income and GDP are expressed in PPP EU27 €.

**Fig. 6:**
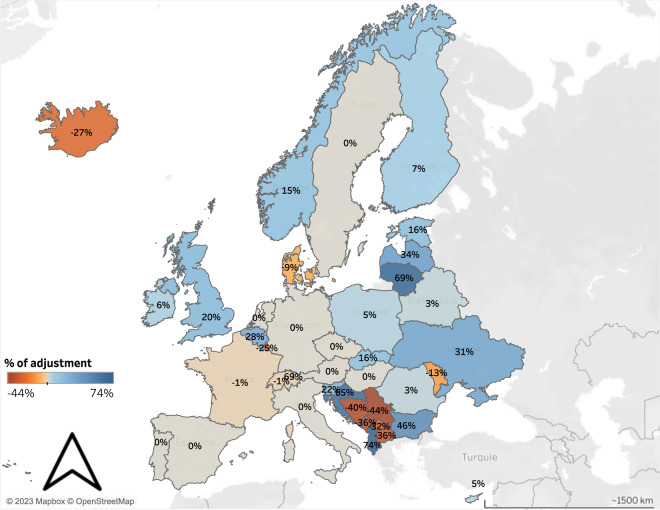
Country average income adjustment for 2015.

To compare the two adjustment methods (estimates / Eurostat ratios vs. GDP / income ratios), we calculated the average adjustment coefficient used in each country (Fig. 6). Income adjustment was relatively low in Southern and Western Europe (values ranged between −1% and 28%). Values were significantly higher in Eastern and Central European countries (values ranged between −44 and 83%). This can be explained by the high quality of NSIs’ income data and of the regressions used to estimate disposable income in Eurostat countries.

For each country, the figure represents the average coefficient used to adjust estimated income to disposable income. Countries with positive values were adjusted upward and conversely for countries with negative values. Countries not represented in the map have not been adjusted either because it was not needed (i.e. when data was directly collected from Eurostat such as in Greece) or because we could not find a country-level source of disposable income.

**Fig. 7:**
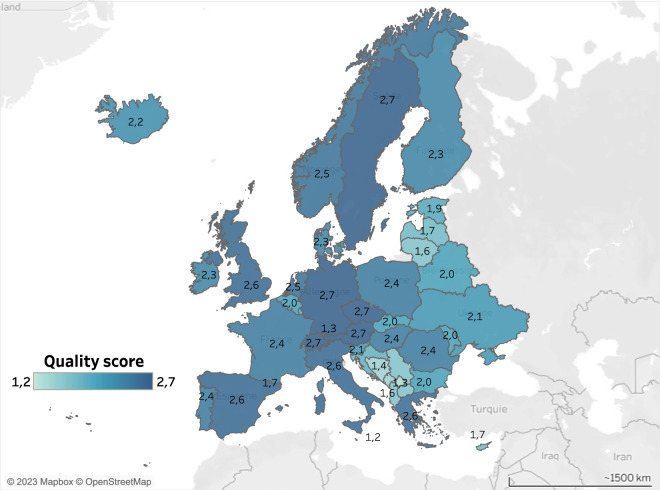
Country weighted-average quality score.

The map of weighted-average quality score revealed a divide between the Balkan and Baltic states and the rest of Europe regarding the quality of data provided by NSI (Fig. 7), the former showing low values for the quality score (mostly below 2) while the latter showed higher values. A radar chart representing the quality score of each country (including for the 6 components separately) is provided in Supplementary Information (Figure S2).

## Usage Notes

This dataset could be used in socio-economic or ecological studies that require multi-year harmonized income data for several European countries. For instance, this dataset could be combined with spatial data about ecosystems or infrastructures to analyze how the exposure to environmental risks or the access to environmental amenities intersect with income inequality. In climate and environmental science, gridded datasets are more widely used by scientists. Further research could focus on the development of methodologies to accurately downscale the vectorial dataset produced in this study into a high-resolution gridded dataset.

### Supplementary information

Supplementary Information

## Data Availability

The code supporting the analyses is accessible in the Zenodo through Gitlab repository^11^, under the GNU Affero General Public License v3.0.
