# Supplementary material for: Harmonized disposable income dataset for Europe at subnational level
Source: Sci Data. 2024 Mar 21;11:308. doi: 10.1038/s41597-024-03138-x (PMC10957931; doi:10.1038/s41597-024-03138-x)
Supplement: Supplementary file 1 — Supplementary Information [file 41597_2024_3138_MOESM1_ESM.pdf]

Supplementary Information:
Harmonized disposable income dataset for Europe at subnational level

Mehdi Mikou[1,2], Améline Vallet[1,2], Céline Guivarch[1]

1. Université Paris-Saclay, AgroParisTech, CNRS, Ecole des Ponts ParisTech, Cirad, EHESS, UMR CIRED, 94130, Nogent-sur-Marne, France.

2. Université Paris-Saclay, CNRS, AgroParisTech, Ecologie Systématique et Evolution, 91190, Gif-sur-Yvette, France.

This Supplementary Information document contains additional information to complement the manuscript. We first provide a country-level description of the harmonization procedure with the sources used to create this dataset. We then present a map of the boundaries of the administrative units to let users appreciate the resolution of the dataset. Finally, we present a country-level decomposition of the 6 components of the quality score.

Table of Contents

1. **Country-level estimation and adjustment procedures**
2. **Resolution of the dataset**
3. **Country quality score by subcomponent**

1. Country-level estimation and adjustment procedures

The table S1 describes the main steps to convert collected income to disposable income for each of the 42 countries in this study. It provides information regarding how disposable income was estimated. If it was estimated through taxation, the column "Tax rates" describes the rate used and the source of the taxation scheme is also provided. In the case where disposable income was estimated using a linear regression, the columns "Number of units" and "R^2" inform on the number of NUTS units used in the regression along the coefficient of determination R^2. It also describes the adjustment methodology used in each country. Steps and links to download input data (income, socioeconomic indicators) can be found in country-level description files and within country Jupyter notebooks contained in the Zenodo repository[1]. * highlights countries for which input data was obtained from third-parties.

Table S1: Country-level description of the estimation and adjustment procedures

**Country**	Income indicator	National Statistical Institute/Third-party source	Name of download income variable and download url	Estimation of disposable income (M2)	Number of units	R^2	Tax rates	Adjusting disposable income (M3)	Source (Tax rates)
**Albania**	Household consumption expenditure	INSTAT (Institute of Statistics) [2]	Total monthly Income	Taxation	-	-	13% on marginal income over 30,000 and below 50,000 Lek	GDP to Income ratio	[3]
**Andorra**	Mean income	Estadistica Andorra[4]	Mean Income	-	-	-	-	Country level	-
**Austria***	Net income	Organisation for Economic Cooperation and Development (OECD) [5]	-	Linear regression	9	0.87	-	Eurostat NUTS2	-
**Belarus**	Gross income	International Cooperation and Statistics Dissemination Department National Statistical Committee Republic of Belarus [6]	All types of income	Taxation	-	-	Flat tax of 13%	GDP to Income ratio	[7]
**Belgium***	Total net taxable income	STATBEL[8]	Statistique fiscale des revenus par secteur statistique	Linear regression	15	0.98	-	Eurostat NUTS2	-
**Bosnia-Herzegovina**	Average net and gross earnings	Agency for Statistics of Bosnia and Herzegovina [9]	Average monthly net and gross earnings of persons in employment	-	-	-	-	GDP to Income ratio	-

**Bulgaria**	Total gross income per capita	NSI Bulgaria[10]	Total gross income per capita in Lev	-	-	-	-	Eurostat NUTS2	-
**Croatia**	Net earnings	Croatian Bureau of Statistics [11]	Employment and Wages - Review by counties	-	-	-	-	Eurostat NUTS2	-
**Cyprus**	Net Income	Statistical Service of the Republic of Cyprus [12]	HOUSEHOLD BUDGET SURVEY - MEAN ANNUAL INCOME, 2015/2016	-	-	-	-	Eurostat NUTS2	-
**Czech Republic***	Average disposable income	Czech Statistical Office [13]	Average disposable income	-	-	-	-	Eurostat NUTS2	-
**Denmark***	Equivalized disposable income	The Economic Council of the Labour Movement [14]	-	-	-	-	-	Eurostat NUTS2	-
**Deutschland**	Tax income	INKAR [15]	Datenbankdownload	Linear regression	401	0.7	-	NUTS3	-
**Estonia**	Gross earnings	Statistics Estonia [16]	ST004: AVERAGE MONTHLY GROSS INCOME PER EMPLOYEE	Linear regression	22	0.84	-	Eurostat NUTS2	-
**Finland**	Average disposable income	Statistics Finland [17]	Inhabitants' disposable monetary income	-	-	-	-	Eurostat NUTS2	-
**France**	Median equivalised disposable income	Institut national de la statistique et des études économiques [18]	Revenus et pauvreté des ménages	Linear regression	15	0.68	-	Eurostat NUTS2	-
**Greece**	Average disposable income	Eurostat [19]	Disposable income of private households by NUTS 2 regions	-	-	-	-	Eurostat NUTS2	-
**Hungary***	Total taxable income	Organisation for Economic Cooperation and Development (OECD) [5]	-	Linear regression	8	0.85	-	Eurostat NUTS2	-
**Iceland**	Average disposable income	Statistics Iceland[20]	Income by municipalities and sex 1990-2022	-	-	-	-	-	-
**Ireland**	Median gross income	Central Statistics Office[21]	Household Median Gross income - IIA01	Linear regression	37	0.97	-	Eurostat NUTS2	-
**Italy***	Taxable income	Dipartimento Finanze[22]	Redditi e principali variabili Irpef su base comunale	Linear regression	21	0.99	-	Eurostat NUTS2	-

**Kosovo**	Net average wage	Kosovo agency of statistics[23]	Net average monthly wage	-	-	-	-	GDP to Income ratio	-
**Latvia**	Mean disposable income	Official Statistics of Latvia[24]	Households disposable income in regions	-	-	-	-	Eurostat NUTS2	-
**Liechtenstein**	Median gross wage	Statistics office of Liechtenstein[25]	Gross monthly wage by municipality of residence and sex	-	-	-	-	Country level	-
**Lithuania**	Average disposable income	Statistics Lithuania[26]	Average disposable income per month	-	-	-	-	Eurostat NUTS2	-
**Luxembourg**	Median salary	National institute for statistics and economic studies of Luxembourg[27]	Indice socio-économique par commune	-	-	-	-	Eurostat NUTS2	-
**Macedonia**	Disposable income	State Statistical Office of the Republic of North Macedonia[28]	Total disposable household income by types	-	-	-	-	GDP to Income ratio	-
**Malta**	Disposable income	National Statistics Office[29]	Total Disposable Income	-	-	-	-	-	-
**Moldova**	Disposable income	National Bureau of Statistics of the Republic of Moldova[30]	Disposable incomes of population by sources of income and by statistical regions	-	-	-	-	GDP to Income ratio	-
**Montenegro**	Mean equivalised disposable income	Statistical Office of Montenegro - MONSTAT[31]	Survey on Income and Living Conditions	-	-	-	-	GDP to Income ratio	-
**Netherlands**	Average equivalized income	Statistics Netherlands[32]	Inkomen van personen; persoonskenmerken, regio (indeling 2020)	Linear regression	12	0.93	-	Eurostat NUTS2	-
**Norway**	Median after-tax income	Statistics Norway[33]	06944: Household income, by type of household	-	-	-	-	Eurostat NUTS2	-
**Poland**	Average gross wages and salaries	Statistics Poland[34]	WAGES AND SALARIES AND SOCIAL SECURITY BENEFITS	Linear regression	324	0.81	-	Eurostat NUTS2	-
**Portugal**	Gross income less income tax	Instituto Nacional de Estatística – Portugal (Statistics Portugal) [35]	Indicators of Declared Gross Income	Linear regression	11	0.64	-	Eurostat NUTS2	-

**Romania**	Average net earnings	The National Institute of Statistics ROMANIA[36]	Average monthly nominal net earnings	Linear regression	13	0.86	-	Eurostat NUTS2	-
**Serbia**	Income in money	The Statistical Office of the Republic of Serbia (SORS) [37]	Household income in money and in kind, by statistical territorial units (NSTU)	Taxation	-	-	Flat tax rate between 10 and 15% (depending on the year)	GDP to Income ratio	[38]
**Slovak Republic**	Disposable income	Statistical office of the Slovak Republic[39]	Equivalised household income - average income by regions [ps3805rr]	-	-	-	-	Eurostat NUTS2	-
**Slovenia**	Net income	Statistical Office of the Republic of Slovenia[40]	Net income received by the population, municipalities, Slovenia, annually	-	-	-	-	Eurostat NUTS2	-
**Spain**	Net Income	Instituto National de Estadistica (INE) [41]	Mean and median income indicators	Linear regression	19	0.93	-	Eurostat NUTS2	-
**Sweden**	Disposable income	Statistics Sweden[42]	Disposable income of households (ESA2010) by region (LAU2)	-	-	-	-	Eurostat NUTS2	-
**Switzerland**	Net income	Swiss federal authorities[43]	Répartition des revenus des personnes physiques	Taxation	-	-	Progressive taxation based on religious affiliation, income level, household composition and canton	Country level	[44]
**Ukraine**	Disposable income	State Statistics Service of Ukraine [45]	Household income and expenditure	-	-	-	-	GDP to Income ratio	-
**United Kingdom**	Gross disposable income	Office for National Statistics [46]	Regional gross disposable household income: local authorities by NUTS1 region	-	-	-	-	-	-

2. Resolution of the dataset

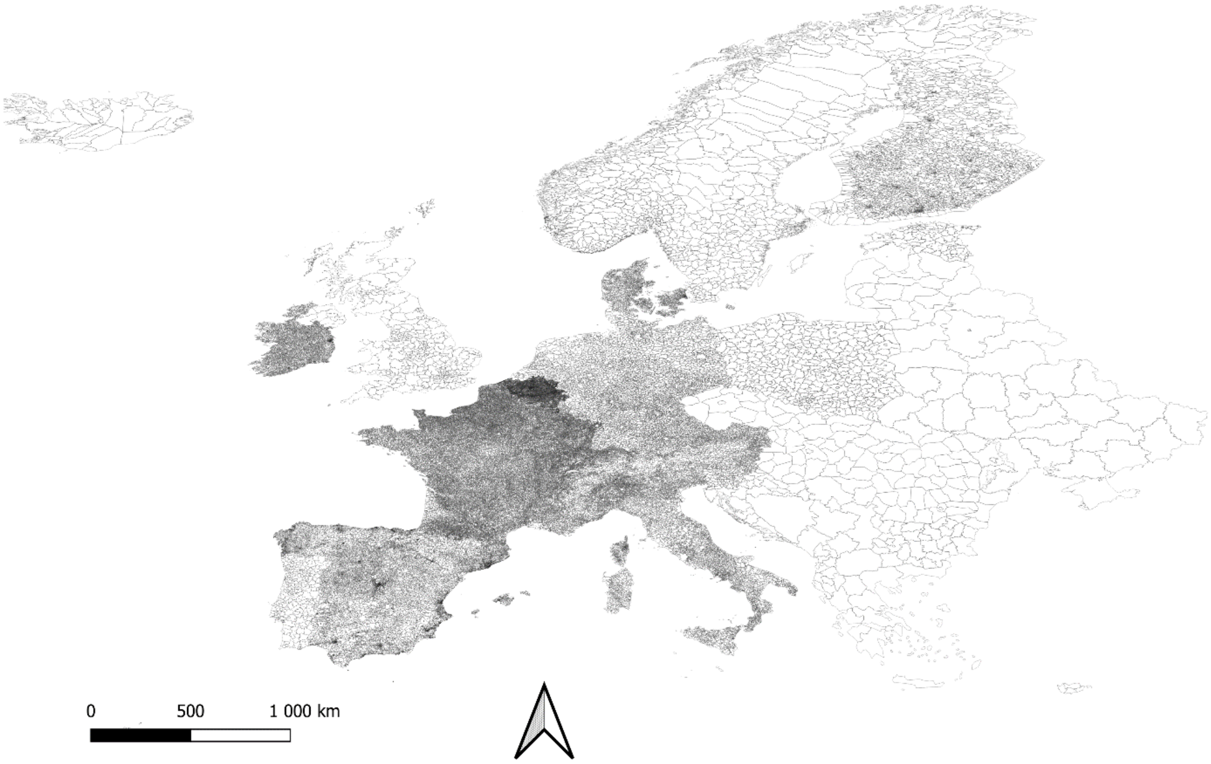

Figure S1: Boundaries of administrative units

3. Country quality score by subcomponent

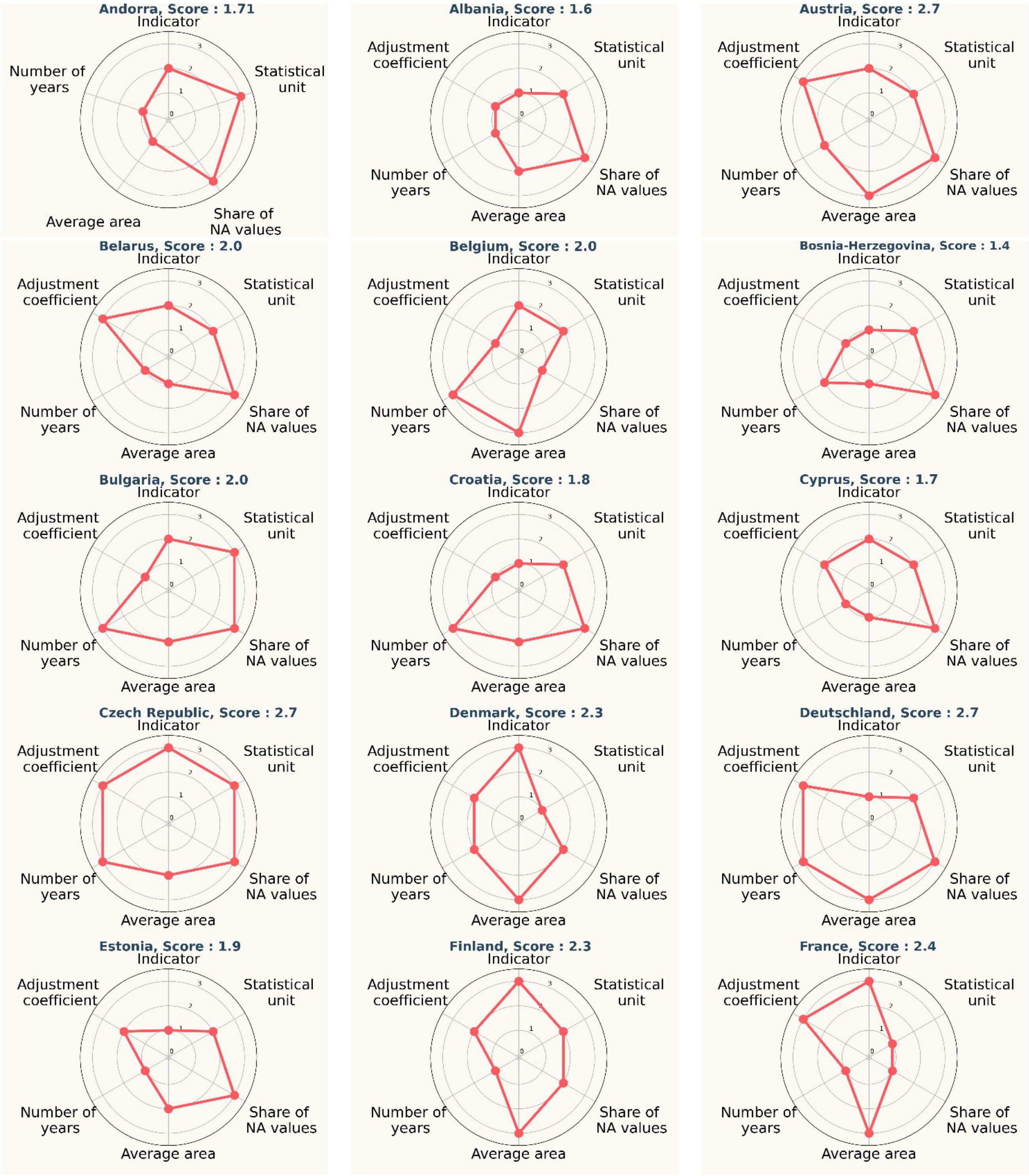

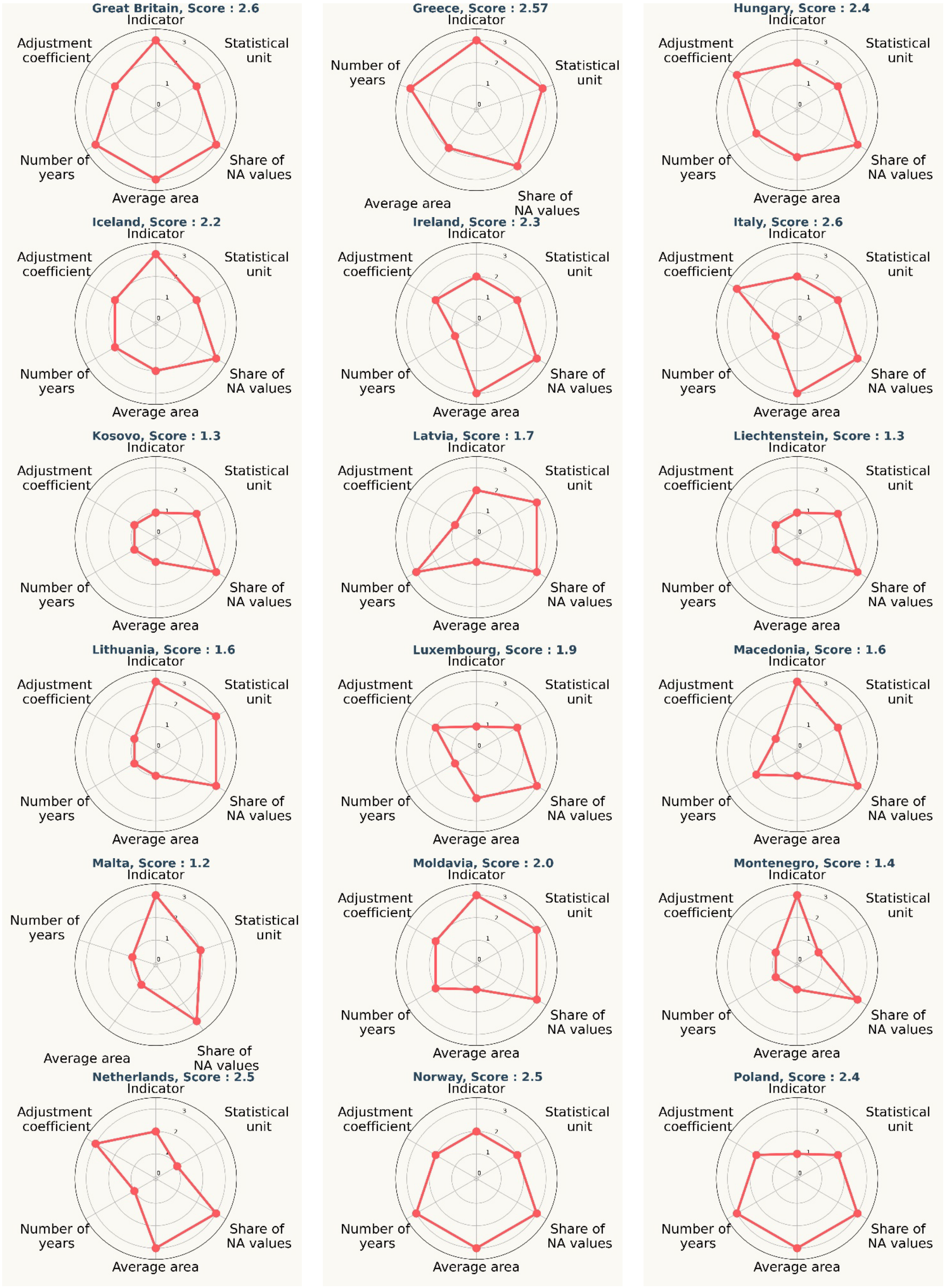
Great Britain, Score : 2.6
Greece, Score : 2.57
Hungary, Score : 2.4
Iceland, Score : 2.2
Ireland, Score : 2.3
Italy, Score : 2.6
Kosovo, Score : 1.3
Latvia, Score : 1.7
Liechtenstein, Score : 1.3
Lithuania, Score : 1.6
Luxembourg, Score : 1.9
Macedonia, Score : 1.6
Malta, Score : 1.2
Moldavia, Score : 2.0
Montenegro, Score : 1.4
Netherlands, Score : 2.5
Norway, Score : 2.5
Poland, Score : 2.4
Indicator
Statistical unit
Share of NA values
Average area
Number of years
Adjustment coefficient

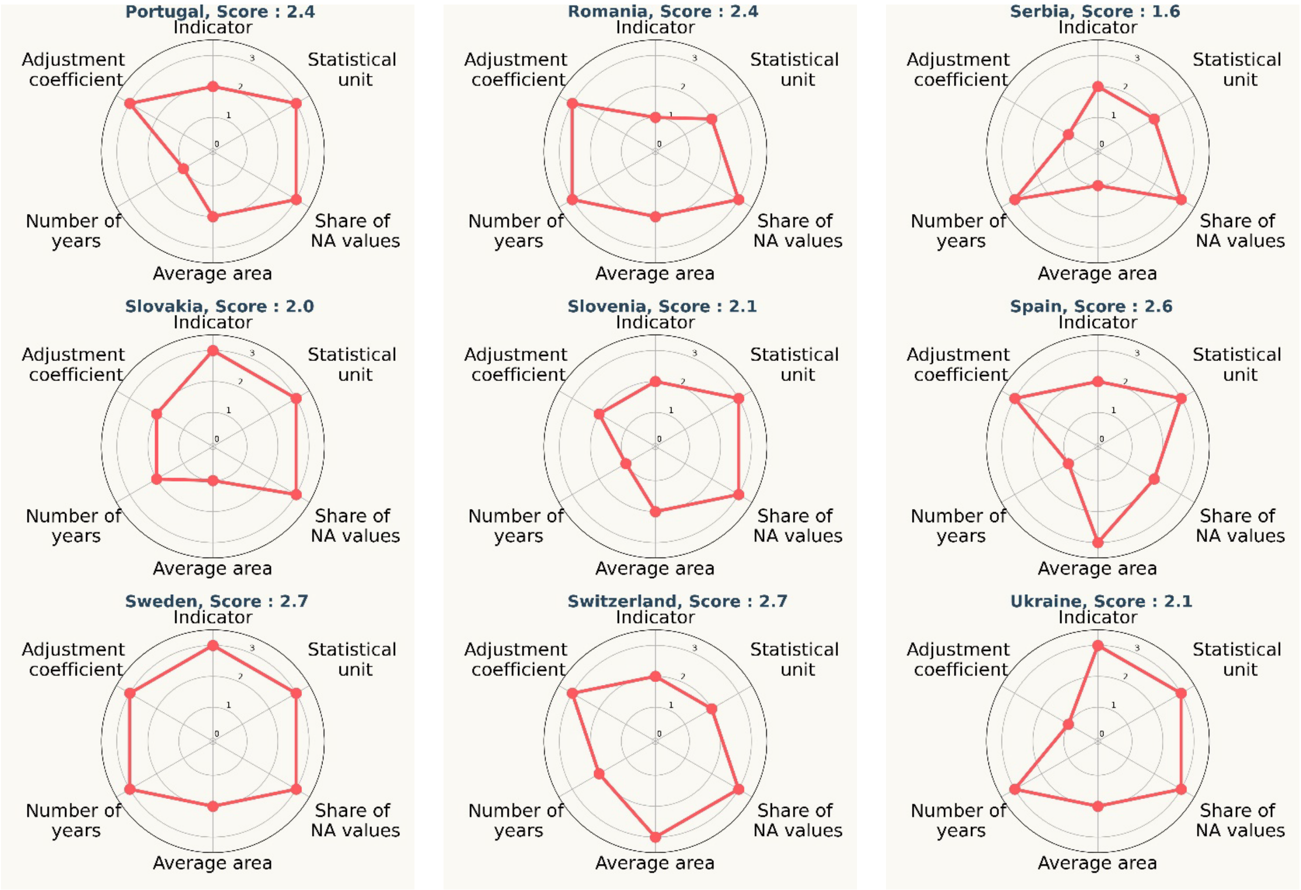

Figure S2: Country-level data quality score

References

1. Mikou, M. Replication code for the publication 'Harmonized disposable income dataset for Europe at subnational level'. Zenodo https://doi.org/10.5281/zenodo.10259732 (2023).
2. INSTAT (Institute of Statistics). INSTAT (Institute of Statistics). *PxWeb* https://www.instat.gov.al.
3. Albania - Individual - Taxes on personal income. https://taxsummaries.pwc.com/albania/individual/taxes-on-personal-income.
4. Departament d'Estadística del Govern d'Andorra. https://www.estadistica.ad/portal/apps/sites/#/estadistica-ca/.
5. Koenigs, S., Vindics, A. & Diaz Ramirez, M. The geography of income inequalities in OECD countries : Evidence from national register data.
6. International Cooperation and Statistics Dissemination Department National Statistical Committee Republic of Belarus. National statistical committee of The Republic of Belarus. http://dataportal.belstat.gov.by/Indicators/Preview?key=175855.
7. Belarus Personal Income Tax Rate. https://tradingeconomics.com/belarus/personal-income-tax-rate.
8. Statbel, l'office belge de statistique | Statbel. https://statbel.fgov.be/fr.
9. Agency for Statistics of Bosnia and Herzegovina. https://bhas.gov.ba/.
10. National statistical institute Bulgaria. National statistical institute. https://infostat.nsi.bg/.
11. Croation Bureau of Statistics. Statistics in line. *Državni zavod za statistiku* https://dzs.gov.hr/en.
12. Statistical Service of the Republic of Cyprus. Republic of Cyprus, Statistical Service. https://www.cystat.gov.cy/.
13. Czech Statistical Office. Statistics VDB. https://vdb.czso.cz/.
14. The Economic Council of the Labour Movement. The Economic Council of the Labour Movement | Arbejderbevægelsens Erhvervsråd. https://www.ae.dk/the-economic-council-of-the-labour-movement.

15. INKAR. INKAR - BBSR. https://www.inkar.de/?fbclid=IwAR1ItvyA6_ZYvlgPvz327stvQcJL_97RSfZm_yyvA4GwABGSX_7wQa-zi6U.
16. Statistics Estonia. https://www.stat.ee/et.
17. Statistics Finland. 3. Inhabitants’ disposable monetary income by Postal code area, Information and Year. *PxWeb* https://pxdata.stat.fi:443/PxWebPxWeb/pxweb/en/Postinumeroalueittainen_avoin_tieto/Postinumeroalueittainen_avoin_tieto__uusin/paavo_pxt_12f1.px/.
18. Accueil - Insee - Institut national de la statistique et des études économiques. https://www.insee.fr/fr/accueil.
19. Statistics | Eurostat. https://ec.europa.eu/eurostat/databrowser/view/NAMA_10R_2HHINC/default/table?lang=EN.
20. Statistics Iceland. *Statistics Iceland* https://statice.is.
21. Home - CSO - Central Statistics Office. https://www.cso.ie/en/index.html.
22. Dipartimento delle Finanze. *Dipartimento Finanze* https://www.finanze.gov.it/opencms/it/.
23. Kosovo agency of statistics. Kosovo agency of statistics. https://rks-gov.net/.
24. Official Statistics of Latvia. Official statistics of Latvia. *Oficiālās statistikas portāls* https://stat.gov.lv/en.
25. Statistics office of Liechtenstein. Statistics office of Liechtenstein. *PX-Web* https://etab.llv.li:443/PXWebPXWeb/pxweb/en/eTab/eTab__Employment and earnings__Wages/291.041e.px/.
26. Statistics Lithuania. Statistics Lithuania. https://vda.lrv.lt/en/.
27. National institute for statistics and economic studies of Luxembourg. National institute for statistics and economic studies of Luxembourg. https://statistiques.public.lu/fr.htm (2022).
28. State Statistical Office of the Republic of North Macedonia. State Statistical Office of the Republic of North Macedonia. *PxWeb* https://www.stat.gov.mk/Default_en.aspx.

29. NSO Malta. National Statistics Office. *NSO Malta* https://nso.gov.mt/.
30. National Bureau of Statistics of the Republic of Moldova. NATIONAL BUREAU OF STATISTICS of the Republic of Moldova. *PxWeb* https://statistica.gov.md/en.
31. Statistical Office of Montenegro - MONSTAT. Statistical Office of Montenegro - MONSTAT. http://www.monstat.org/eng/.
32. Statistics Netherlands. Statistics Netherlands. https://www.cbs.nl/en-gb/.
33. Statistics Norway. Statistics Norway. *SSB* https://www.ssb.no/en.
34. Statistics Poland. Statistics Poland. https://stat.gov.pl/en/.
35. Instituto Nacional de Estatística – Portugal (Statistics Portugal). Statistics Portugal - Web Portal. https://www.ine.pt/xportal/xmain?xpid=INE&xpgid=ine_main.
36. The National Institute of Statistics ROMANIA. TEMPO Online. http://statistici.insse.ro:8077/tempo-online/#/pages/tables/insse-table.
37. The Statistical Office of the Republic of Serbia (SORS). The Statistical Office of the Republic of Serbia (SORS). https://www.stat.gov.rs/en-us/.
38. Serbia - Individual - Income determination. https://taxsummaries.pwc.com/serbia/individual/income-determination.
39. Statistical office of the Slovak Republic. Statistical office of the Slovak Republic. https://slovak.statistics.sk.
40. Statistical Office of the Republic of Slovenia. Statistical Office of the Republic of Slovenia. *PX-Web* https://pxweb.stat.si/SiStat/en.
41. Instituto National de Estadistica (INE). Instituto National de Estadistica (INE). *INE* https://ine.es/.
42. Statistics Sweden. Statistics Sweden. *Statistikdatabasen* https://www.scb.se/en/.
43. AFC, A. fédérale des contributions. Statistiques sur l'impôt fédéral direct. https://www.estv.admin.ch/estv/fr/home/die-estv/steuerstatistiken-estv/allgemeine-steuerstatistiken/direkte-bundessteuer.html.
44. Federal Tax Administration - FTA. https://www.estv.admin.ch/estv/en/home.html.

45. State Statistics Service of Ukraine. State Statistics Service of Ukraine. https://www.ukrstat.gov.ua/.

46. Office for National Statistics. Office for National Statistics. https://www.ons.gov.uk/.
